# Mechanisms of chromosomal instability (CIN) tolerance in aggressive tumors: surviving the genomic chaos

**DOI:** 10.1007/s10577-023-09724-w

**Published:** 2023-04-14

**Authors:** Brittiny Dhital, Veronica Rodriguez-Bravo

**Affiliations:** 1 Department of Biochemistry and Molecular Biology, Mayo Clinic, Rochester, MN USA; 2 Department of Urology, Mayo Clinic, Rochester, MN USA; 3 Thomas Jefferson University Graduate School, Philadelphia, PA USA; 4 Mayo Clinic Graduate School of Biomedical Sciences, Rochester, MN USA

**Keywords:** CIN, Cancer, Metastasis, Therapy resistance, CIN adaptation, CIN tumors therapeutic vulnerabilities

## Abstract

Chromosomal instability (CIN) is a pervasive feature of human cancers involved in tumor initiation and progression and which is found elevated in metastatic stages. CIN can provide survival and adaptation advantages to human cancers. However, too much of a good thing may come at a high cost for tumor cells as excessive degree of CIN-induced chromosomal aberrations can be detrimental for cancer cell survival and proliferation. Thus, aggressive tumors adapt to cope with ongoing CIN and most likely develop unique susceptibilities that can be their Achilles’ heel. Determining the differences between the tumor-promoting and tumor-suppressing effects of CIN at the molecular level has become one of the most exciting and challenging aspects in cancer biology. In this review, we summarized the state of knowledge regarding the mechanisms reported to contribute to the adaptation and perpetuation of aggressive tumor cells carrying CIN. The use of genomics, molecular biology, and imaging techniques is significantly enhancing the understanding of the intricate mechanisms involved in the generation of and adaptation to CIN in experimental models and patients, which were not possible to observe decades ago. The current and future research opportunities provided by these advanced techniques will facilitate the repositioning of CIN exploitation as a feasible therapeutic opportunity and valuable biomarker for several types of human cancers.

## Introduction

Chromosomal aberrations in animal cells and tissues have been observed since the nineteenth century, as reported by Hansemann and “the Boveris” (Marcella O’Grady and Theodor), and suggested a connection to cancer and organismal development defects (Boveri 2008; Hansemann 1890; Satzinger 2008).

**Fig. 1:**
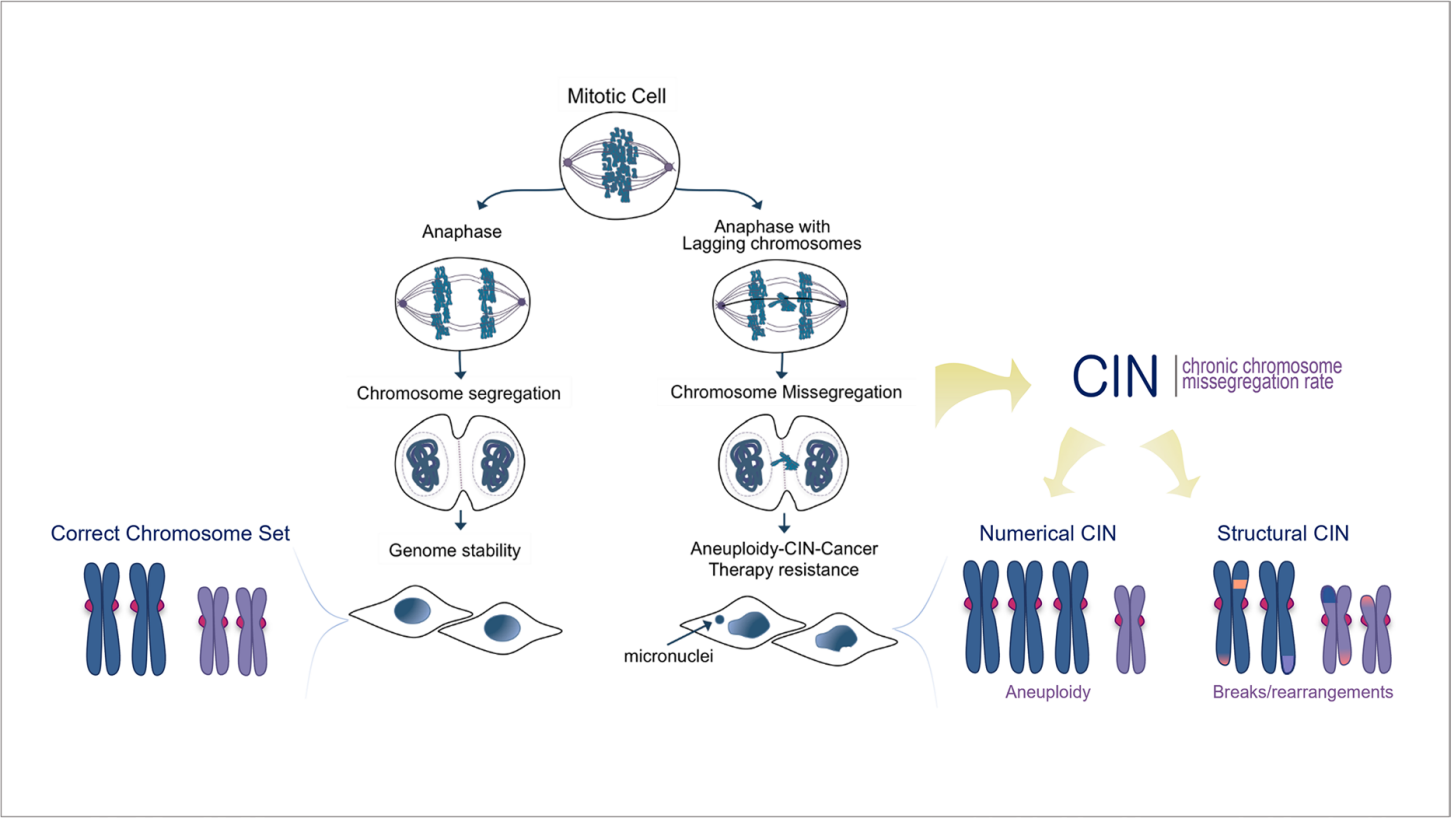
Diagram depicting human cells undergoing normal versus aberrant chromosome segregation that leads to CIN. Normal anaphase and cell division allows high fidelity separation of chromosomes between daughter cells and maintenance of genome stability and normal chromosomal content. Lagging DNA during anaphase can result in chromosome missegregation during cell division. Chromosomal instability (CIN) is the persistent rate of chromosome segregation errors every cell division in a cell population, CIN can be numerical which leads to unbalanced chromosome numbers or Aneuploidy, or structural in which results in damaged, broken, or rearranged chromosomes. CIN fuels the accumulation of further genomic changes promoting cancer progression, metastasis, and therapy resistance

Since then, chromosomal defects have been established as pervasive alterations in tumor cells intrinsically linked to critical failure during chromosome segregation and cellular division, impacting genome balance, cell viability, organism development, and cancer. Under normal conditions, each step of the cell cycle is tightly regulated to ensure mitotic fidelity. But failure of these surveillance mechanisms can compromise error-free chromosome segregation and result in persistent chromosomal defects being passed to cell progeny, including chromosomal instability (CIN), aneuploidy, or chromothripsis (Funk et al. 2016; Thompson et al. 2010; Torres et al. 2008). CIN has traditionally been described as an ongoing chromosomal content gain or loss every cell division. This terminology can lead to consider that CIN is synonymous with aneuploidy, in which the cell chromosome number deviates from the standard euploid set. On the contrary, CIN encompasses an array of chromosomal defects, of which aneuploidy is one of the possible outcomes. Another common misconception is to consider CIN as a static phenomenon where a single event leads to a one-time alteration of a cell’s chromosomes that can then be passively transmitted to future daughter cells through successive divisions. This is far from reality as CIN is a dynamic ongoing process in which cells continually missegregate chromosomal DNA every time they divide, with the potential to acquire multiple types of aberrations, resulting in enriched genetic diversity with each subsequent mitosis (Fig. 1).

More recently, updated definitions have improved the previous simplistic depiction and offer a more cogent characterization of the complexities surrounding chromosomal aberrations. Presently, CIN is being defined as chromosomal alterations that can be categorized as either numerical, when whole-chromosome missegregation is involved, or structural, when only affecting partial fragments of chromosomes (Geigl et al. 2008). Numerical whole-chromosome CIN (W-CIN) can lead to aneuploidy, which is the wrong number of whole chromosomes in cells, whereas structural CIN (S-CIN) refers to alterations in fragments of individual chromosomes and can result in insertions, deletions, inversions, translocations, and as an extreme case, chromothripsis, which refers to the shattering and complex random reassembly of an entire missegregated chromosome at micronuclei (Cortés-Ciriano et al. 2020; Crasta et al. 2012; Zhang et al. 2015). Different mechanisms known to date that can lead to the generation of whole and structural CIN mostly include, but are not limited to, chromosomal segregation errors during mitosis, DNA replication errors and stress, telomere dysfunction, lack of correction of merotelic kinetochore-microtubule interactions, centrosome amplification, dysfunctionality of nuclear pores, biased chromosomal positioning and segregation, centromere integrity, or chromothripsis, all of which have been comprehensively covered by others and will not be discussed in detail here (Bailey and Murnane 2006; Bakhoum and Swanton 2014; Barra and Fachinetti 2018; Burrell et al. 2013; Cimini 2008; Crasta et al. 2012; Davoli and de Lange 2012; Davoli et al. 2010; Fouladi et al. 2000; Ganem et al. 2009; Gordon et al. 2012; Gregan et al. 2011; Holland and Cleveland 2009; Klaasen et al. 2022; Lingle et al. 2002; Ly and Cleveland 2017; Maciejowski and de Lange 2017; Rodriguez-Bravo et al. 2014; Sabatier et al. 2005; Thompson et al. 2010; Thompson and Compton 2011; Venkatesan et al. 2021; Worrall et al. 2018; Zhang et al. 2015). The focus of this review is to address the consequences and changes taking place in chromosomally unstable tumor cells, with the aim of discussing the potential of exploiting CIN adaptation mechanisms to better guide future treatment strategies. Notably, different types of chromosomal defects derived from CIN can coexist or be independent of each other. For example, aneuploidy, one of the possible outcomes of CIN, is not necessarily indicative of an unstable genome but denotes an erroneous ploidy that gets perpetuated without further changes in a cell population. In comparison, CIN that induces chromothripsis may promote “hyper instability” phenotypes affecting certain regions of the genome or whole chromosomes, leading to the formation of a highly complex genome structure. The mechanisms triggered by different types of chromosomal aberrations and the potential adaptations in cancer cells may share common aspects but also differ. In this review, we discussed up-to-date literature covering CIN and aneuploidy, which have been closely linked and extensively studied. We have attempted to establish the underlying cell-intrinsic and -extrinsic mechanisms associated with these abnormalities, wherever possible. However, future research is necessary to elucidate and more clearly delineate the commonalities and differences between cell responses to CIN, aneuploidy, and other chromosomal alterations.

## CIN in healthy cells

As expected, CIN and the derived aneuploidy are not well tolerated in healthy cells, and the effects can be devastating. In the case of aneuploid cells, even a single change in the chromosome number can have a tremendous impact on cell fitness and viability (Siegel and Amon 2012; Torres et al. 2008; Williams et al. 2008). During development, aneuploidy can seriously affect the embryo viability and result in early miscarriage in utero, death shortly after birth (Ambartsumyan and Clark 2008; Fragouli et al. 2013; Nagaoka et al. 2012; Soler et al. 2017; van den Berg et al. 2012) different degrees of developmental abnormalities in children, and chromosomal disorders, including Down syndrome (chromosome 21 trisomy) (Dierssen et al. 2009). After development, the occurrence of somatic CIN is associated with cellular senescence, tissue aging, infertility (Andriani et al. 2016; Baker et al. 2004; Ly et al. 2000; Macedo et al. 2018), and neurodegenerative diseases, including Alzheimer’s (Granic et al. 2010; Iourov et al. 2009).

CIN and aneuploidy can have catastrophic consequences for viability, and they are heavily selected against (Pfau et al. 2016). As a result, cells have developed robust autonomous regulatory mechanisms during cellular division to prevent the accumulation and persistence of chromosomal aberrations. If these safeguards fail, cell-extrinsic detection systems involving immunoresponses are in place to initiate appropriate processes leading to the elimination of cells carrying aberrant chromosomal content. Thus, apart from cell-autonomous pathways involved in maintaining the fidelity of DNA replication and segregation during mitosis, cellular non-autonomous surveillance systems have been recently reported to be involved in clearance of cells carrying imbalanced chromosomal content (aneuploid) by the immune system, including natural killer (NK) cells (Santaguida et al. 2017; Wang et al. 2021). In addition, CIN triggers an inflammatory response through activation of the cGAS-STING pathway, an specialized antiviral mechanism that detects double-stranded DNA spilled into the cytosol from ruptured micronuclei containing defective chromosomal content (Mackenzie et al. 2017; Motwani et al. 2019) leading to the subsequent downstream activation of the type I interferon pathway (Galluzzi et al. 2018; Sun et al. 2013) to initiate innate and adaptive immune responses that clear the damaged cell, a process that can be hijacked by cancer cells as further discussed below (Bakhoum et al. 2018). Therefore, CIN-derived missegregated chromosomal DNA in micronuclei can be a source of proinflammatory signals that should trigger the elimination of highly genomic unstable cells. These safety mechanisms protect healthy cell populations from developing persistent CIN under normal conditions. However, in certain cases, such as cancer, these defense mechanisms are often insufficient, altered, or hijacked, which can allow the propagation of CIN.

## Revisiting the CIN paradox in malignant cancer

CIN is a well-documented hallmark in human cancers occurring in as high as 80% of tumors (Bakhoum and Compton 2012; Lengauer et al. 1998; Weaver and Cleveland 2006). It is particularly associated with more aggressive subtypes, such as metastases or cancers of unknown origin (CUP) (Olivier et al. 2021), and has been linked to therapy resistance, tumor evolution, and adaptability (Bakhoum and Cantley 2018; Drews et al. 2022; Ippolito et al. 2021; Lee et al. 2011; Nguyen et al. 2022; Salgueiro et al. 2020; Sansregret et al. 2018; Santaguida and Amon 2015; Swanton et al. 2009; Tamura et al. 2020; Tijhuis et al. 2019; Watkins et al. 2020). If non-cancerous cells are unable to survive with CIN, it raises the question of why CIN is so prevalent in tumors. Although CIN is selected against owing to its impact on cellular fitness, this is not as clear in the case of cancer cells. Recent research has suggested that the effects of CIN and derived aneuploidy on tumors may depend on disease and tissue context, as well as on the levels of CIN. As a result, CIN can have varying outcomes on tumors, ranging from tumor promotion to tumor suppression (Ben-David and Amon 2020; Birkbak et al. 2011; de Cárcer et al. 2018; Funk et al. 2016; Hoevenaar et al. 2020).

CIN has been shown to promote tumorigenesis in mice models and to provide genetic diversity and adaptive advantages in tumor cell populations (González-Loyola et al. 2015; Sansregret and Swanton 2017; Sansregret et al. 2018; Sotillo et al. 2007). However, studies using mice models engineered to decrease the dose of the centromere protein CENP-E (CENP-E + / −), which displayed both ongoing moderate CIN due to chromosome missegregation events and aneuploidies, indicate a context specific effect for tumor formation in selective animal tissues. Interestingly, the study found tumorigenesis was promoted in tissues with low baseline levels of aneuploidy; conversely, tumor formation was suppressed in tissues prone to high chromosome missegregation, such as the liver, indicating CIN and aneuploidy can both promote and suppress tumorigenesis depending on the context (Weaver et al. 2007).

A clinical study comparing the outcomes of cancer patients with different CIN levels in their tumors indicated that patients whose tumors had intermediate levels of CIN displayed more aggressive disease and had a worse prognosis than those with tumors carrying low or very high CIN (Birkbak et al. 2011; Jamal-Hanjani et al. 2015; Roylance et al. 2011). These observations were validated in an independent pan-cancer study evaluating tumor fraction of the genome altered (FGA) and patient survival (Andor et al. 2016). These clinical data indicate that even though cancer cells may be able to persist with some intermediate CIN levels in aggressive carcinomas, they may be intolerant to excessive CIN. This also suggests that there must be some CIN thresholds that tumor cells can tolerate and, when surpassed, may be extremely detrimental for tumor survival and progression. These ideas were already suggested decades ago by Lengauer and Vogelstein’s teams, who proposed that the right levels of CIN may allow clonal populations to bypass selection barriers if those CIN level did not kill cells in the first place (Cahill et al. 1999).

Additional studies from the Weaver group using mice models and MEF cells further verified that the induction of low levels of chromosomal missegregation promote tumorigenesis (CENP-E + / −  alone), whereas higher rates of chromosome missegregation (CENP-E + / − ; Mad2 + / − double heterozygous mice) have the opposite effect and are tumor suppressive (Funk et al. 2016; Silk et al. 2013). These studies confirmed that CIN and derived aneuploidy can act as oncogenic triggers but also as tumor suppressors (Weaver et al. 2007). Thus, although certain low levels of CIN may promote “healthy” inter- and intratumor heterogeneity, boost genetic diversity, and confer adaptive advantages that endow cancer cells to persist adverse changes in their environment, for example, survive the selective pressures imposed by anti-cancer therapies, having too much of a good thing may be a potential weakness and excessive chromosomal chaos levels can greatly reduce cancer cell fitness.

All these observations in normal and tumors tissues have helped to better contextualize the well-known CIN and aneuploidy paradox, that postulates that CIN can be both detrimental and advantageous and conclude that CIN-persistent cells existing in tumors can themselves be susceptible to excessively high levels of chromosomal aberrations. Further supporting this idea, a recent study has demonstrated that high-CIN therapy-refractory metastatic tumors develop transcriptionally driven vulnerabilities that can be targeted to induce lethal levels of chromosomal aberrations in tumors (Dhital et al. 2023).

However, many questions remain to be answered to completely understand the underlying mechanisms modulating CIN levels in tumor cells at different disease stage, tissues, and therapy exposure and their potential therapeutic implications. These include the following: what is the “just right” amount of CIN tolerable by a tumor cell? What are the fitness penalties imposed by different classes of CIN (numerical versus structural) or chromosomal aberrations in each tumor cell context? The future of the CIN field should address these questions at the molecular level in experimental models and using clinical patient data. Despite the significant preference for CIN accumulation in cancer cells, the actual rates of CIN in human cancer are just starting to be comprehensibly studied at the genomic level in patients, indicating a clear correlation between higher CIN levels and metastases (Dhital et al. 2023; Drews et al. 2022; Nguyen et al. 2022). It will be paramount for future studies to be able to match clinical findings to molecular mechanisms of CIN induction and modulation using experimental approaches in different tumor cell models.

However, tumor cells do not exist in voids, and in addition to the cell-intrinsic mechanisms described above, it is essential to also consider the contributions to CIN and aneuploidy of the tumor microenvironment and associated inflammatory responses. In this regard, several seminal studies have unveiled that CIN-high tumors instigate chronic inflammatory responses via the cGAS-STING pathway and non-canonical NF-kB activation that mediate invasion and metastasis (Bakhoum and Cantley 2018; Bakhoum et al. 2018). In addition, chromosomally unstable tumors have been shown to develop a survival dependency on the cGAS-STING pathway, a vulnerability that can be targeted by blocking the interleukin 6 signaling (Hong et al. 2022). These findings underscore the microenvironment surrounding the tumors as a relevant scenario that integrates signals from cancer cells carrying chromosomal defects. This novel area of study has also generated a new paradox since in normal cells inflammation signals promote clearance of aneuploid cells by immune cells (NK cells) (Santaguida et al. 2017), whereas studies in tumors indicate that aneuploidy favors immunosuppression (Davoli et al. 2017). Similarly, chronic inflammatory signals from CIN tumors promote metastasis instead of preventing tumor progression or tumor cell elimination (Bakhoum and Cantley 2018; Tijhuis et al. 2019). Thus, it will be important to better define the inflammatory mechanisms associated with acute versus sustained aneuploidy and CIN, as well as to identify any differences between normal and cancer cells and tissues.

Overall, clinical and experimental evidence indicates that as tumors progress and become more aggressive, they continue to accumulate higher CIN levels without losing their potential for proliferation and survival. This raises the question of how cancer cells conserve their precarious balance between CIN-derived benefits and toxicity. Furthermore, the mechanisms by which tumors adapt to ongoing CIN and promote their continued survival, progression, and metastatic dissemination while bypassing tissue and therapy barriers remains largely unknown. Hence, the remainder of this review is dedicated to discussing the mechanisms by which tumors can cope with higher levels of CIN, with a focus on the potential to exploit these dependencies to improve patient outcomes.

## Mechanisms of CIN attenuation in cancer

How tumor cells cope with ongoing CIN and what determines the right levels for survival remain poorly understood aspects of cancer cell biology. In this context, a few studies have begun to illuminate the potential implications of different cell-autonomous mechanisms in the adaptation to CIN. Classically, *TP53* loss has been associated with the perpetuation of CIN in patient tumors (Burrell et al. 2013). Mechanistically, p53 can induce G1 cell cycle arrest after chromosome missegregation (Ohashi et al. 2015; Soto et al. 2017; Thompson and Compton 2008, 2010) and restrain aneuploidy-induced tumorigenesis in mice in conjunction with the activation of the ATM kinase pathway (Li et al. 2010) or via p21 upregulation (Barboza et al. 2006). These studies indicate that loss of a classic tumor suppressor, like the TP53 gene, is a main factor that releases the brake and allows cell populations with CIN to keep proliferating (Sansregret et al. 2018), suggesting this mechanism may be linked to a DNA damage response triggered by chromosome missegragations that induce breaks in the DNA. As many human tumors display a high mutation rate in p53, this may explain why CIN cells escape initial elimination. However, not every tumor carries loss-of-function p53 mutations (Soussi and Wiman 2007), suggesting the existence of alternative pathways that restrain CIN perpetuation in tissues. This idea is further supported by studies in transgenic mice demonstrating that high CIN induces comparable apoptosis and tumor suppression levels in p53 null and p53 wild-type animals (Funk et al. 2021). Similarly, another study reported that loss-of-function mutations in the BCL9L gene, particularly in aneuploid colorectal cancer cells, promote their propagation by reducing caspase-2 and attenuating cell death, irrespective of p53 status, after chromosome segregation errors (López-García et al. 2017). Likewise, recent research suggested that aneuploid cancer cells that accumulate DNA damage can ensure their survival through the RAF/MEK/ERK pathway in a p53-independent way (Zerbib et al. 2023).

Nevertheless, beyond chromosomal stability and/or ploidy checkpoint barriers leading to cell cycle arrest and/or death after DNA missegregation in mitosis, other mechanisms are emerging through which tumor cells alleviate the levels of CIN to make them compatible with survival, previously referred to as maintaining “just right” amounts of genome chaos to keep going (Cahill et al. 1999). In this regard, downregulation of the anaphase-promoting complex (APC/C) components have been shown to allow tumor cells to limit excessive CIN after weakening the spindle checkpoint (SAC) with an Mps1 kinase inhibitor (Sansregret and Swanton 2017; Wild et al. 2016). The authors of the study used this system to screen for factors whose downregulation allowed cells to extend their time in mitosis and avoid extreme chromosome missegregation. The APC/C is an E2 ubiquitin ligase that is inhibited by the SAC until all chromosomes are properly attached and bi-oriented between the two spindle poles, and ready to allow anaphase onset and finish chromosome segregation. Previous studies have demonstrated that when the function of the APC/C complex is tuned down using inhibitors, this extends the duration of mitosis and allows cells to improve their chromosome segregation fidelity and reduce CIN (Rodriguez-Bravo et al. 2014; Zeng et al. 2010). Notably, the authors identified mutations in APC/C subunits in different percentages of patient tumors, indicating the clinical relevance of this mechanism. The question remains about how prevalent this mechanism is in diverse tumor types and at different stages of tumor development and treatment, and how to define potential targetable strategies to capitalize on these mutations. Similarly, determining whether this is an adaptation response to CIN alone or also to aneuploidy or other chromosomal aberrations would be informative.

Another study from Swanton’s group also suggested that early acquisition and tolerance to whole-genome doubling (WGD) events in colorectal cancer may precede aneuploidy and CIN tolerance, although the specific molecular mechanisms remain undefined (Dewhurst et al. 2014). Recent studies have revealed that the viability of cells that undergo WGD and become tetraploid depends on the mitotic kinesin *KIF18A* (Quinton et al. 2021). This dependency is also present in chromosomally unstable and aneuploid tumor cells (Cohen-Sharir et al. 2021; Marquis et al. 2021), and provides specificity for potential therapeutic avenues to target tumor cells bearing chromosomal aberrations without affecting healthy cells. Tetraploidy is considered an early event in tumorigenesis; however, in healthy tissues, it is highly selected against by tumor suppressors, including *TP53* and the Hippo pathways (Andreassen et al. 2001; Ganem et al. 2014). It would be interesting to further evaluate whether similar dependencies generated early during tumor initiation in tetraploid cells can play a role during disease progression and in later stages including metastatic tumors.

In this regard, the initial clinical correlations indicating CIN to be higher in highly aggressive tumors have been confirmed in pan-cancer genome sequencing studies using FGA as a proxy, as well as using gene signatures and analysis of tissue samples to score missegregation events in anaphases (Bakhoum et al. 2018; Carter et al. 2006; Dhital et al. 2023; Drews et al. 2022; Miller et al. 2020; Nguyen et al. 2022). All these studies indicate that CIN is higher in metastatic disease stages and is particularly enriched in specific tumor types. However, the causes or consequences of CIN and other chromosomal aberrations, such as aneuploidy or chromothripsis, and their fitness penalties and adaptations remain to be elucidated at the molecular level. It remains paradoxical that higher CIN is associated with more aggressive tumors when they are already disseminated and colonizing other organs, once more underscoring the dynamic nature of CIN accumulation and potential tolerance mechanisms that allow cells with chaotic genomes to continue proliferating or even restrain their levels of CIN, all of which could be context-dependent and change from early onset to disease progression. Why and how metastatic tumors can survive with high aberrant chromosome content, and the impact of different therapeutic approaches remain interesting areas of investigation that require more molecular understanding. Chromosomal instability has been shown to promote metastasis by inducing an epithelial-mesenchymal transition (EMT) phenotypic switch in cells while modulating the TME with a chronic inflammatory cGAS-STING-dependent response that contributes to metastatic phenotype persistence (Bakhoum and Cantley 2018). Numerous questions remain unaddressed to better understand the evolution of these mechanisms over time during disease progression and the potential differences inflicted by diverse types of genome aberrations including changes in chromosomes numbers, alterations affecting chromosomes’ structure or mixed phenotypes. Additionally, it is equally important to understand the contribution of various TME cells and the effects of different therapies on the metastatic sites and niches of high-CIN tumors. For instance, recent findings suggest that the accumulation of unfolded proteins in aneuploid cells induces changes in macrophages and T cells, which promote an immunosuppressive TME (Xian et al. 2021).

We should also consider the impact of chromosome number and structural changes on the proteome and transcriptome of a tumor cell, as they may also lead to potential adaptability mechanisms. For example, aneuploidy induces proteotoxic stress and transcriptional changes in cells (Oromendia et al. 2012; Sheltzer et al. 2012). Thus, it is easy to speculate that hijacking the protein quality control pathways by tumor cells may help alleviate the lack of fitness from CIN-induced dysregulation of the proteome (Torres et al. 2007). Whether these mechanisms are also used to tolerate ongoing CIN remains to be determined. Protein toxicity and gene expression changes as a consequence of aneuploidy and gene copy number alterations (CNAs), and the adaptation to them, have been reviewed comprehensively before, which include mutations observed to increase activity of the proteosome pathway to alleviate proteotoxic stress in aneuploid cells (Torres et al. 2010, 2007), ribosomal loss, ribosomal protein changes (Baker and Montagna 2022; Terhorst et al. 2020), and dosage compensations to adjust gene expression levels in some instances (Kojima and Cimini 2019). The mechanisms developed in aneuploid cells to compensate for increased protein and mRNA production are being further characterized at the molecular level. For example, a recent study found that aneuploid cells, both in cancer cell lines and tumor samples, increase RNA and protein degradation to cope with excessive transcriptional activity and proteotoxic stress (Ippolito et al. 2023). Due to the connexion between protein stress responses and ribosomal changes, it is plausible to consider the potential role of ribosome heterogeneity (Shi et al. 2017) or ribosome collision resolution (Wu et al. 2020) in the adaptation to aneuploidy in tumors. Whether these mechanisms exist or cooperate, and to what extent in response to ongoing CIN in cells, remains to be elucidated.

A recent study has revealed that metastatic therapy-resistant prostate cancer cells undergo transcriptional reprogramming to adapt to persistently elevated CIN by upregulating mitotic fidelity pathways which restrain accumulation of lethal levels of chromosomal errors. This study emphasizes the role of cell rewiring at the chromatin level as a potentially relevant mechanism for adapting to ongoing CIN in metastatic therapy-refractory tumors, and shows that targeting this axis is a feasible strategy to selectively target them by exacerbating their chromosome missegregation rate over the tolerable threshold (Dhital et al. 2023). This concept of upregulation of genome integrity pathways’ to tune down excessive levels of chromosomal abnormalities, which would compromise cell fitness and proliferation, is gaining more momentum. For example, a very recent study indicates that aneuploid cells undergo replication stress which results in CIN and in a diverse outcome of karyotypic complexities and cell fates in their progeny. Of note, in this context, aneuploid/CIN cells that are able to sustain a proliferative status are shown to turn up DNA repair pathways (Garribba et al. 2023). Remarkably, another study has reported cancer-specific epigenetic vulnerabilities in tumor cells with underlying genomic instability that makes them sensitive to targeting the acetyltransferase complex MLS, which leads to exacerbation of under-replicated DNA, CIN, and extreme levels of aneuploidy (Monserrat et al. 2021). Building on this concept, it has been shown that epigenetic changes affecting DNA methylation correlate with CIN and aneuploidy in colorectal cancers (Frigola et al. 2005; Rodriguez et al. 2006). This work was further advanced recently by a study revealing that missegregated chromosomal DNA trapped in micronuclei disrupts histone post-translational modification homeostasis, leading to altered chromatin accessibility and epigenetic interactions that can fuel even more tumor cell adaptability and plasticity (Agustinus et al. 2022). This phenomenon could be linked to the observed gain in epigenetic memory traits driven by oncogenic long non-coding RNAs (lncRNAs) binding at fragile chromatin sites in cancer cells (Arunkumar et al. 2022). Interestingly, in the case of highly aggressive tumors with very poor prognosis, such as CUPs, in which there is a lack of biomarkers and biological signatures allowing proper categorization, CIN is a unique hallmark that can be considered as a trait correlating with the most poorly differentiated tumors, very unfavorable prognosis, and unpredictable metastatic patterns (Olivier et al. 2021). Although it has been suggested there could be epigenetic features unique to CUPs, it remains undetermined whether future epigenomic studies will help to better define CUP tumor epigenetic traits under a “biological signature” correlating well with CIN and whether it can be used as a study model for CIN adaptation. Overall, it would be interesting to better understand the implications of other transcriptional regulation and epigenetics pathways in the modulation and adaptation to CIN and aneuploidy in tumors. This is especially relevant, considering that the driving events of aggressiveness in advanced disease stages, such as therapy resistance and phenotypic switches, have been proposed to be partly owing to non-genetic changes, mostly related to cell rewiring and epigenetic plasticity events (Carceles-Cordon et al. 2020; Huang 2021; Marine et al. 2020; Pisco and Huang 2015; Vendramin et al. 2021).

**Fig. 2:**
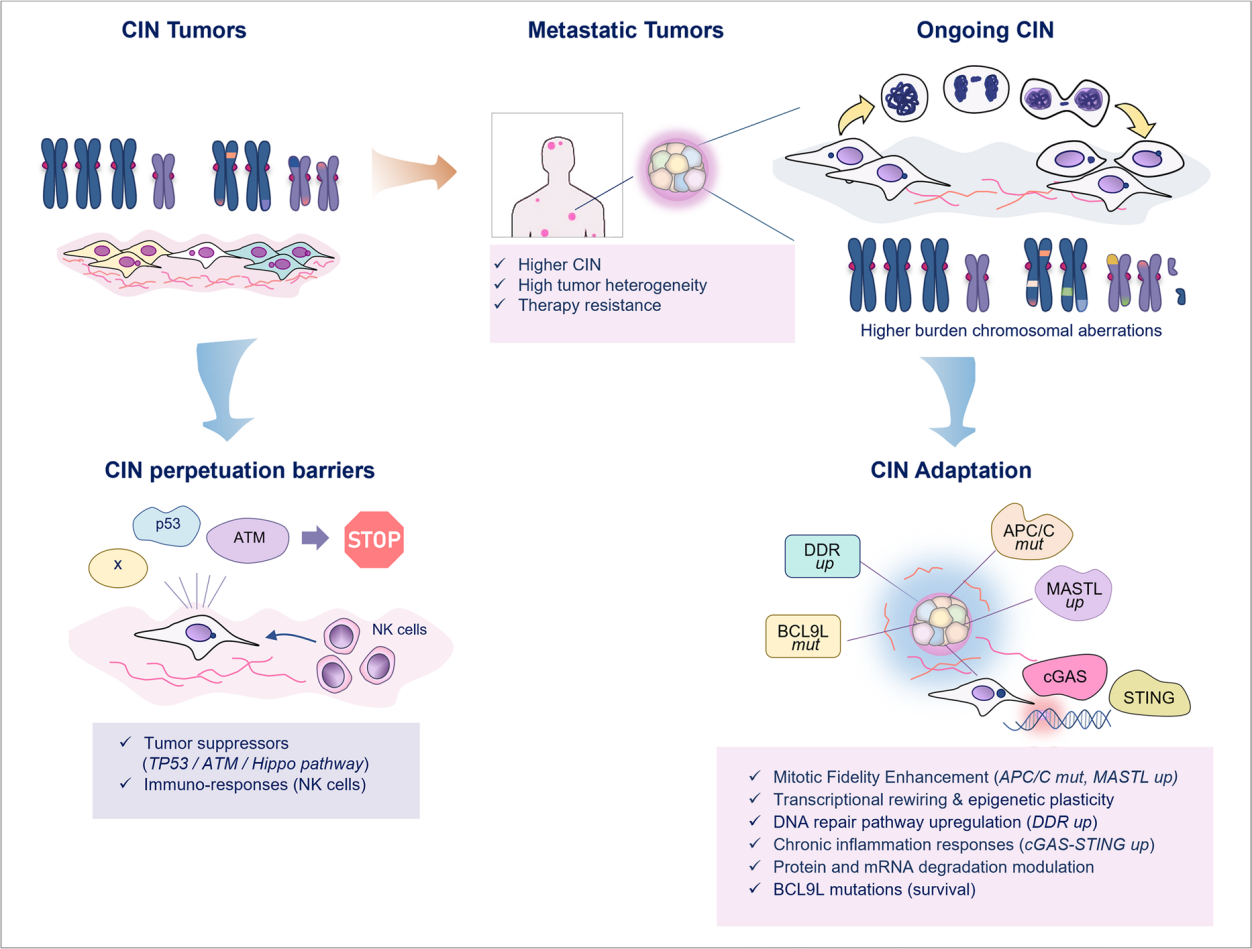
CIN proliferation barriers and mechanisms of adaptation in tumor cells. The diagram depicts reported mechanisms in the literature by which CIN tumor cell perpetuation can be prevented as well as the mechanisms of CIN adaptation in aggressive tumors. The classic tumor suppressor pathways are illustrated as potential roadblock for CIN cell continued proliferation. These pathways include but are not restricted to p53, Atm, and the Hippo pathway. Likewise, aneuploid cells can be eliminated by tumor cell extrinsic mechanisms involving NK cells. However, regardless of tumor suppressor status, metastatic tumors display increased CIN that correlates with high tumor heterogeneity and therapy resistance. Several mechanisms of adaptation are depicted including the reported finding of APC/C mutations that enhance fidelity of mitosis, transcriptional rewiring and upregulation of mitotic fidelity pathways controlled by MASTL kinase, upregulation of the cGAS-STING pathway in response to high CIN in metastasis, pro-survival pathways promoted by BCL9L mutations, and epigenetic reprogramming or upregulation of DNA damage repair (DDR). These and other emerging pathways continue to be heavily investigated in cancer

In conclusion, experimental evidence in cell models, genetically engineered mice, and patient data urges further investigation of the myriad mechanisms that may be utilized by tumor cells to overcome penalties associated with high chromosomal aberration burden (Fig. 2).

It is also paramount to investigate the molecular underpinnings of CIN and aneuploidy adaptation in cancers considering the inherent variability across different tumor types and disease context (e.g., primary vs. disseminated tumors), the impact of therapeutic intervention, and the role of cell-intrinsic and cell-extrinsic mechanisms employed by tumors with varied intermediate to high levels of CIN. The biology underlying these mechanisms may be the key to determining potential new therapeutic interventions directed towards targeting these unique cancer vulnerabilities.

## Therapeutic opportunities to exploit CIN adaptation in cancer

Targeting aneuploid and CIN tumor cells has been proposed as a potential therapeutic strategy (Bakhoum and Compton 2012). However, the main limitation has been the determination of actionable molecular pathways to attack tumor cells that have CIN or aneuploidy selectively to attempt to define “unique vulnerabilities” that are essential particularly for tumor cells. We have summarized above some of the main mechanisms that drive CIN accumulation and most pertinent for this review, the recent literature linking specific “pro-tumor” mechanisms to tolerate and/or eliminate tumor suppressor-driven proliferation barriers of CIN cells.

Although preventing CIN accumulation or stopping CIN cells from perpetuating early on would be ideal, the detection of early CIN cells and lesions is challenging. In addition, after tumorigenic events are initiated, tumors may already be locally advanced or systemically disseminated at diagnosis and, consequently, carry many more genomic alterations and/or already display elevated CIN levels. Thus, it is feasible to consider exploiting the dependencies that tumors develop during progression to survive CIN. This idea is not new, as it has been suggested that harnessing the tumor-detrimental effects of excessive CIN in cancer cells by targeting mitosis using SAC inhibitors enhances the effect of chemotherapy (Funk et al. 2016; Janssen et al. 2011; Kops et al. 2004, 2005; Lee et al. 2016). Similarly, tumor cells with elevated numerical CIN are more susceptible to radiation therapy (Bakhoum et al. 2015). It can be argued that in some of these studies, essential kinases, such as the SAC master regulator Mps1, were being targeted, and that the resulting deleterious effects would be also induced in the healthy cell population of the human body, precluding specificity against CIN or aneuploid persistent tumor cells. However, more recent studies have demonstrated that aneuploid cells and cells that undergo WGD can be selectively targeted via Mps1 kinase inhibition owing to their increased vulnerability to the exacerbated CIN levels (Cohen-Sharir et al. 2021; Quinton et al. 2021). This suggests that it is feasible to design therapeutic approaches that may allow for more selective elimination of CIN tumor cells while diminishing potential toxicities in healthy tissues. Nonetheless, it should be noted that exploiting CIN in cancer cells inducing intolerable levels as a possible therapeutic strategy may induce secondary genome aberrations that could lead to toxicities or secondary tumors in the future, as occurs with some systemic therapies frequently used in cancer treatments currently. In the case of Mps1, a potent inhibitor (CFI-402257) is currently being tested in the clinical setting (Schöffski et al. 2022) for aggressive advanced solid tumors in clinical trials (NCT05251714, NCT05601440). Most notably, in this clinical context, Mps1 inhibition has been shown to be well tolerated in patients (Hilton et al. 2022) and has recently received FDA fast-track designation for the treatment of adult patients with ER + /HER2 − advanced breast cancer after progression to CDK4/6 inhibitors and hormone therapy.

Numerous mitotic genes are essential and embryonically lethal when completely knocked out. However, despite their essentiality in organismal development, many pan-essential genes are currently either targeted in cancer therapy or being studied for this purpose. This is primarily based on the principle of targeting functional dependencies and differential and/or combinatorial vulnerabilities that are specific to cancer cells using inhibitors rather than complete genetic ablation (such as when using constitutive knockout mice or human cell lines). This is clearly demonstrated in the case of Cdk4/Cdk6, two essential cell cycle genes, that when deleted in combination (double KO) result in death at late stages of embryonic development (Malumbres et al. 2004), and yet Cdk4/Cdk6 dual inhibition has become a standard of care for many breast cancer patients, and preclinical data suggest it will also be the case for many other tumor types (Goel et al. 2022; Salvador-Barbero et al. 2020). Similarly, another example of therapeutically targeting cell cycle essential genes in the context of specific functional vulnerabilities of cancer cells identified a specific dependency on CCNE1 (cyclin E) upregulation/amplification in tumor cells, which can be selectively targeted using inhibitors of PKMYT1 (a. k. a Myt1), an essential mitotic kinase (Gallo et al. 2022). Finally, recent studies on metastatic prostate cancer have also proposed targeting high-CIN therapy-refractory tumors, elevating tumor chromosomal aberration to lethal levels by inhibiting the mitotic fidelity kinase MASTL (Dhital et al. 2023). These two abovementioned studies suggest that the upregulation of the cell cycle and mitotic machinery observed in certain tumors may not just only be a cause of initial CIN generation or pure proliferation; but at least in the case of advanced aggressive tumors already sufficiently replete with CIN, the mitotic fidelity mechanisms may have adapted to be more efficient creating new strong dependencies and unique vulnerabilities that can be therapeutically exploited in cancer cells.

The use of drugs to disrupt mitosis as a cancer treatment is not a novel concept and has been widely used for decades. Microtubule-targeting agents (MTAs) are a potent treatment option that induce intratumor high chromosome missegregation in cancer (Zasadil et al. 2014), and the main stay of chemotherapeutic treatments for several cancers. However, their efficacy is limited by their non-specificity, as all cells, whether cancerous or otherwise normal, can be affected, leading to adverse toxicities (Dumontet and Jordan 2010). Within this context, there is an increasing consensus in the literature that more studies are needed to further investigate mitotic kinase inhibition in cancer therapeutics and to improve their therapeutic index. Thus, the enthusiasm to further investigate therapeutic targeting of pan-essential genes for cancer therapy, including cell cycle and mitotic fidelity genes, continues as has been recently discussed in other reviews (Chang et al. 2021; Suski et al. 2021), which suggested that, moving forward, partial loss-of-function studies, rather than only complete loss (shRNA or CRISPRi vs. CRISPR knock outs), should be included as a potential better strategy to identify differential effects across cancer models and uncover “selectively lethal” cancer targets.

Beyond the cell cycle armamentarium to target chromosomal aberration adaptation, recent studies suggest harnessing CIN and aneuploidy by inhibiting other signaling angles. For example, it has been recently shown that cancer cells that are chromosomally unstable are dependent on the cGAS–STING–STAT3 and non-canonical NF-κB pathway inflammatory responses to survive and that blocking IL-6 signaling targets aggressive high-CIN breast cancer cells’ growth (Hong et al. 2022). This study supports the idea that targeting the cGAS-STING-dependent inflammatory signaling cascade generated as a consequence of CIN, using IL-6 blockers or STING agonists (Decout et al. 2021; Scott 2017), could be a good therapeutic strategy for CIN tumors. Based on this suggestion, it would be also interesting to test the validity of standard of care treatments including radiotherapy (Cosper et al. 2022), DNA damaging agents (Cheung-Ong et al. 2013), endocrine therapy in hormone-driven cancers (Risbridger et al. 2010), and others, in combination with cGAS-STING blockage, as well as to evaluate recently FDA-approved agents in the genome integrity space such as PARP or ATR inhibitors, within the context of synthetic lethality and beyond (Mullard 2022).

Thus, the connection between CIN, inflammation, and tumor immunity are increasingly becoming more studied as comprehensively reviewed by others (Bakhoum and Cantley 2018; Tijhuis et al. 2019). Nonetheless, more studies are required to better understand how tumors with high CIN or high aneuploidy impact the TME and modulate acute and chronic inflammatory signals and the consequences for immune responses and efficacy of systemic and targeted therapies, including immunotherapy. Similarly, future research should dissect potential differences between tumors with different degrees of chromosomal aberrations as well as diverse types of defects accumulated, from structural CIN, numerical CIN, aneuploidy, or chromothripsis, to better direct future therapies and biomarkers predictors of response.

In this regard, and based on the most recent literature, aneuploidy and CIN may instigate different inflammation and immune responses, suggesting diverse associated mechanisms that require further investigation (Davoli et al. 2017; Santaguida et al. 2017). In this space, recent studies and ongoing research efforts are being directed to determine the suitability of using genomic measures of aneuploidy to predict immunotherapy response. It has been proposed that using tumor aneuploidy scores, which measure the fraction of chromosome arms with copy number alterations, and/or fraction of the genome altered (FGA), which has been proposed as a proxy of tumor CIN (Nguyen et al. 2022), could be a good approach to predict immunotherapy response in cancer patients with tumors carrying low tumor mutational burden (TMB) (Chang et al. 2022; Spurr et al. 2022b). This would represent a remarkable opportunity to exploit aneuploidy and CIN in the biomarker space. Tumor mutational burden has been established as a biomarker predicting immunotherapy response of tumors when TMB itself is high, but there is a big portion of cancer patients whose tumors do not fall in this category and who could potentially benefit from complementary predictor tools. In this regard, a recent study has reported that high tumor aneuploidy provides improved responses to combined immune checkpoint blockade and radiation therapy in non-small cell lung cancer (Spurr et al. 2022a). Further investigation is required to determine the mechanisms that may promote or suppress immunotherapy responses in the context of aneuploid or CIN tumors, and the value of measuring aneuploidy and/or FGA/CIN as biomarkers of therapy response in bigger patient sample sizes and across tumor types. In addition, it would be necessary to study the power of these new biomarkers in different contexts of a disease course, such as for example before or after therapies and considering if tumors acquired resistance to previous treatments. Nevertheless, we should anticipate that aneuploidy and/or CIN may expose unique new avenues for therapeutic interventions in cancer and their scoring could become useful predictors of response to different types of systemic therapy strategies.

## Concluding remarks

Recent genomic analysis of tumor tissues and experimental cell models have demonstrated that the incidence of CIN is higher in advanced aggressive tumors. It is now clear that low levels of CIN can promote tumor progression, but exacerbated CIN levels can also be detrimental for tumor cells. Today, we are closer to better understanding this paradox at the molecular level with the goal of utilizing that knowledge for clinical interventions. Despite the prevalence of CIN in cancer and its association with worse patient outcomes, the primary use of CIN has been limited to classic pathological classification and, more recently, to the determination of genetic heterogeneity in tumors, rather than as a pure diagnostic tool with therapeutic value. Recent genomic sequencing approaches are beginning to improve our understanding of CIN as a promoter of tumor evolution and adaptability, as well as helping to classify the levels and subtypes of genomic alterations across all human cancers.

We anticipate that future studies will provide more mechanistic insights and potential new applications in both the biomarker and cancer therapeutic space. For example, because of its implications in drug resistance and patient survival, determination of CIN levels and characteristics by applying standard cytogenetics and/or more advanced techniques could be suited as part of routine cancer diagnoses and clinical follow-up rather than simply serving as a classification tool. Such inclusion would greatly benefit patient outcomes and guide clinical treatment programs by stratifying patients based on CIN levels to predict those who would benefit from CIN-based therapies in the future. Although recent in vitro and in vivo studies focusing on exploiting CIN-induced dependencies in cancer cells have shown promise for future therapies, further mechanistic understanding is required to disentangle the complex web surrounding tolerance to different types of CIN, both numerical and structural, and to better understand their value in the clinical context of cancer therapeutics.

Questions remain unanswered to have a more holistic understanding of the consequences of CIN increase during tumor progression at the molecular level and the similarities or differences existing with aneuploid tumors. Here, we have discussed the up-to-date evidence from different studies which have started to elucidate tumor mechanisms of genomic chaos adaptation and to unveil opportunities for therapeutic targeting.

## Data Availability

Not applicable.

## Abbreviations

*APC/C*: Anaphase promoter complex/cyclosome
*ATM*: Ataxia telangiectasia mutated
*ATR*: Ataxia telangiectasia and Rad3 related
*BCL9L*: B-cell CLL/lymphoma 9 like
*cGAS*: Cyclic GMP-AMP synthase
*CDK*: Cyclin-dependent kinase
*CCNE1*: Cyclin E1
*CNA*: Copy number alterations
*CIN*: Chromosomal instability
*CUP*: Cancer of unknown origin
*EMT*: Epithelial-mesenchymal transition
*ER*: Estrogen receptor
*ERK*: Extracellular signal-regulated kinase
*FDA*: Federal Drug Administration (USA)
*FGA*: Fraction of the genome altered
*HER2*: Receptor tyrosine-protein kinase erbB-2
*KIF18A*: Kinesin family member 18A
*LncRNA*: Long non-coding RNA
*NK*: Natural killer
*NF-kB*: Nuclear factor kappa-light-chain-enhancer of activated B cells
*MASTL*: Microtubule-associated serine/threonine kinase
*MEK*: Mitogen-activated protein kinase
*Mps1*: Monopolar spindle 1
*MTA*: Microtubule-targeting agents
*PARP*: Poly(ADP-ribose) polymerase
*PKMYT1 (Myt1)*: Membrane-associated tyrosine- and threonine-specific cdc2-inhibitory kinase
*RAF*: Raf-1 proto-oncogene, serine/threonine kinase
*S-CIN*: Structural chromosomal instability
*SAC*: Spindle assembly checkpoint
*STING*: Stimulator of interferon genes
*TME*: Tumor microenvironment
*TP53 (p53)*: Transformation-related protein 53
*W-CIN*: Whole chromosomal instability (numerical CIN)
*WGD*: Whole-genome doubling
